# Recovery of Rare Earth Elements from Different Types of Coal Fly Ash by Direct Bioleaching

**DOI:** 10.3390/ma19132861

**Published:** 2026-07-04

**Authors:** Shulan Shi, Ting Chen, Simeng Ren

**Affiliations:** Key Laboratory of Coal Processing & Efficient Utilization, Ministry of Education, School of Chemical Engineering and Technology, China University of Mining and Technology, Xuzhou 221116, China

**Keywords:** coal fly ash type, rare earth elements, selectivity, occurrence modes

## Abstract

Coal fly ash contains valuable rare earth elements (REEs), making its comprehensive utilization highly significant. In this study, REEs were recovered from two typical types of coal fly ash by *Acidithiobacillus ferrooxidans*, and the influencing factors and leaching mechanisms were explored. The results showed that favorable pulp density for bioleaching was 2% and the bacterial inoculum size was 5%. Among the three tested energy substances, the leaching system with sulfur as the energy source achieved the highest REE leaching efficiency. The Zhunneng coal fly ash bioleaching system had a REE leaching efficiency of 40.41%, which was significantly higher than that of Faer coal fly ash (12.93%). Generated from a pulverized coal furnace, Faer coal fly ash had a spherical shape and smooth surface, differing markedly from the Zhunneng samples from a circulating fluidized bed. Compared with the Zhunneng sample, there are more quartz and mullite in Faer coal fly ash which are refractory to bacterial decomposition. Also, Faer coal fly ash contains more REEs associated with aluminosilicate, while Zhunneng coal fly ash contains more organic/sulfide-bound and acid-soluble REEs. Furthermore, bioleaching demonstrated high selectivity for REEs. This study provides new insights for the green and economic management of coal fly ash.

## 1. Introduction

Coal fly ash (CFA), a byproduct of coal combustion in thermal power plants, is a complex material with significant commercial potential. Primarily composed of amorphous phases, mullite, quartz, hematite, and magnetite [1], coal fly ash features a dense surface network of Si-O-Si and Si-O-Al bonds. This unique structure provides remarkable chemical stability and oxidation resistance [2]. As the primary solid waste from coal combustion, coal fly ash production exceeds 800 million tons annually [3]. In China, the dominant disposal method is landfill, which poses environmental challenges. This method not only requires substantial land areas but also risks air, soil, and water contamination [4]. Notably, coal fly ash contains rare earth elements (REEs), such as Ce, Y, La and Pr [5]. This highlights the potential of coal fly ash as a resource for recovering valuable REEs. However, the REE content in coal fly ash is low, and most REEs are encapsulated within silicate and aluminosilicate matrices [6], limiting the widespread application and exploitation of coal fly ash [7]. With increasing global demand for REEs and advancements in extraction technology, the REEs in coal fly ash have become increasingly viable for recovery [8].

Traditional REE extraction methods are physical and chemical processes that often involve high-temperature alkaline roasting and acid leaching. These methods suffer from high cost, high pollution, and poor selectivity for REEs [9]. In contrast, bioleaching offers a promising sustainable alternative, characterized by lower energy requirements, reduced environmental impact, and potentially lower costs [10]. Bioleaching utilizes biocatalytic reactions mediated by microbial metabolites such as inorganic acids and organic acids to dissolve metals from ores [11]. The extraction process mainly involves acidolysis, complexation, biosorption and bioaccumulation [12], and is mediated by various microorganisms including bacteria, archaea, and fungi [11,13].

In 2001, Seidel & Zimmels [14] investigated aluminum (Al) leaching from coal fly ash using metabolic products (sulfuric acid) from *Thiobacillus thiooxidans*. They found that after three weeks of leaching, the maximum Al leaching efficiency was 25% [14]. Using sulfur-oxidizing microorganisms, 77% Al and 43–79% REEs were extracted from lignite ash [15]. In 2023, Ma [16] conducted a study on REEs bioleaching from coal fly ash using *Aspergillus niger*, which could secrete organic acids, primarily oxalic and citric acids. As a result, the leaching efficiency of REEs was 30.91%, with the leaching efficiency for gadolinium (Gd) reaching 46.63%. Additionally, Park & Liang [17] investigated three microbial species, *Candida bombicola*, *Phanerochaete chrysosporium*, and *Cryptococcus curvatus* for REEs leaching from coal fly ash. Among these, *C. bombicola* demonstrated the best performance, with 64.6% erbium (Er) and 67.7% ytterbium (Yb) being extracted. Collectively, bioleaching shows potential to be a sustainable successor to conventional metal recovery methods. However, REE bioleaching efficiencies vary significantly across studies. Different microbial species are employed in different studies, which leads to different REE extraction efficiencies. Additionally, the property of coal fly ash is also a key factor affecting the bio-extraction of REEs [18], but the underlying mechanisms remain unknown.

*Acidithiobacillus ferrooxidans* is an obligate aerobic, Gram-negative chemoautotrophic bacterium capable of oxidizing ferrous iron, sulfur and sulfide to obtain energy [19]. Its applications range from bioleaching, electronic waste recycling, and sludge treatment [20]. Fan & Lv [3] studied the bioleaching of coal fly ash using *A. ferrooxidans* with pyrite as the energy source. They found that the leaching efficiency of Al peaked at 91.2% on day 12, while the maximum leaching efficiency of cerium (Ce) was 63.4%. Notably, the coal fly ash was pretreated via roasting with Na_2_CO_3_, which converted aluminum-bearing minerals into more leachable forms, thereby enhancing metal extraction. However, jarosite formed during bioleaching with pyrite as an energy source, which limits the dissolution of REEs [3]. Using an energy source without iron may solve the problem, but this approach remains unexplored. Su & Chen [21] employed hydrothermal alkali pretreatment coupled with *Acidithiobacillus thiooxidans* bioleaching to extract REEs from coal fly ash. Hydrothermal alkali treatment enhanced the bioleaching efficiency of V, Sr, Y, La, and Ce. Although alkaline pretreatment of coal fly ash can improve bioleaching efficiency of REEs, it increases processing costs. Moreover, alkaline pretreatment reduces selectivity for REEs, which makes subsequent metal separation and purification more difficult.

*A. ferrooxidans* can fix carbon dioxide [22], and has a high tolerance to toxic metals, making it a key microorganism for developing green and low-carbon industrial processes. In this study, direct bioleaching of coal fly ash by *A. ferrooxidans* was conducted. Bioleaching conditions were optimized, and selectivity to REEs was also investigated. Furthermore, the effects of coal fly ash type on the bioleaching of REEs and the underlying leaching mechanisms were studied. This study provides strong support for the green and economic utilization of coal fly ash.

## 2. Materials and Method

### 2.1. Bacterial Activation

The bacteria strain *Acidithiobacillus ferrooxidans* ATCC 23270 was provided by the Key Laboratory of Biometallurgy of the Ministry of Education of China, Central South University, Changsha, China. The cultivation medium used was 9K medium with the following composition: 3 g/L (NH_4_)_2_SO_4_, 0.1 g/L KCl, 0.5 g/L K_2_HPO_4_, 0.5 g/L MgSO_4_·7 H_2_O, 0.01 g/L Ca(NO_3_)_2_, and 10 g/L FeSO_4_⋅7H_2_O or 10 g/L sulfur powder. The pH was adjusted to 1.8–2.0 with sulfuric acid, and the medium was sterilized by autoclaving at 121 °C for 20 min.

### 2.2. Coal Fly Ash and Pyrite Samples

Coal fly ash samples were obtained from the Zhunneng power station, Jungar, Inner Mongolia, China and Faer power station, Liupanshui, Guizhou, China. The Zhunneng CFA was obtained from a circulating fluidized bed (CFB), whereas the Faer CFA was generated by a pulverized coal furnace (PC). The elemental composition of the two CFA samples was reported by Pan et al. [23] They are mainly composed of SiO_2_ and Al_2_O_3_. The Faer CFA has more Fe_2_O_3_ (12.1%) than the Zhunneng CFA (2.59%). The total REE content is 516.80 ppm for Zhunneng CFA and 520.27 ppm for Faer CFA, respectively. La, Ce, Nd, Pr and Y are the predominant REEs in both samples [23]. Prior to the experiment, a pyrite sample was ground and sieved through a 200-mesh screen.

### 2.3. Bioleaching Experiments

#### 2.3.1. The Impacts of Pulp Density and Inoculum Size on Bioleaching

To establish a leaching system, 0.5 g of elemental sulfur was added as an energy substance to a 150 mL Erlenmeyer flask filled with 50 mL of culture medium, and Zhunneng coal fly ash was used. Three gradients of pulp density were tested at 2%, 5%, 10% (*w*/*v*), with the inoculation amount at 5% (*v*/*v*). A bacterial culture with a cell density of 1.3 × 10^9^ cells/mL was prepared. Three gradients were designed for inoculation amount of bacteria solution: 5%, 10%, 20% (*v*/*v*), at a fixed 2% pulp density. All leaching experiments were conducted for 14 days at 30 °C with a shaking speed 170 rpm. During the leaching process, the pH value, concentration of REEs, and bacterial concentration were monitored every 3 days. Bacterial concentration was determined using a hemacytometer. At the end of leaching, Al concentration was measured. During the bioleaching process, sterilized ultrapure water was added according to the weight loss every 12 h. Leaching systems without bacteria served as blank control groups. Abiotic controls containing sulfuric acid instead of sulfur powder were also set, and the molar concentration of sulfuric acid was the same as that of the sulfur powder (0.31 mol/L). Each experiment was performed in triplicate.

#### 2.3.2. Different Energy Substances

In order to explore the impact of energy source on bioleaching, 0.5 g of pyrite or FeSO_4_ was utilized as an energy substance instead of elemental sulfur, in both the Zhunneng and Faer coal fly ash bioleaching systems. Bioleaching experiments were carried out for 14 days at 2% pulp density and 5% inoculation amount. The leaching efficiencies of REEs and pH dynamics were compared across leaching systems with different energy substances. During bioleaching, *A. ferrooxidans* drives the oxidation of elemental sulfur, pyrite, and ferrous sulfate to produce H_2_SO_4_ and Fe^3+^ [24], as shown in Equations (1)–(3) [3,20,25]. The bio-sourced H_2_SO_4_ participated in the dissolution of REE carriers in CFA, releasing REEs into solution (Equations (4) and (5)).

8H_2_O (l) + FeS_2_ (s) + 14Fe^3+^ (aq) → 15Fe^2+^ (aq) + 2SO_4_^2−^ (aq) + 16H^+^ (aq) (1)

2S^0^ (s) + 3O_2_ (g) + 2H_2_O (l) → 2SO_4_^2−^ (aq) + 4H^+^ (aq) (2)

4Fe^2+^ (aq) + 4H^+^ (aq) + O_2_ (g) → 4Fe^3+^ (aq) + H_2_O (l) (3)

REE_2_O_3_ (s) + 6H^+^ (aq) → 2REE^3+^ (aq) + 3H_2_O (l) (4)

REE_2_(CO_3_)_3_ (s) + 6H^+^ (aq) → REE^3+^ (aq) + 3H_2_O (l) + 3CO_2_ (g) (5)

#### 2.3.3. Faer Coal Fly Ash Bioleaching System

REEs in Faer coal fly ash were leached by *A. ferrooxidans*. The experiment was conducted in a 150 mL Erlenmeyer flask filled with 50 mL basal 9K medium and 0.5 g sulfur powder, under conditions of 5% bacterial inoculum size and 2% pulp density. The systems were incubated in a constant temperature shaker at 30 °C for 14 days, with a shaking speed of 170 rpm. The pH, leaching efficiencies of REEs, and bacteria concentration were monitored every 3 days.

### 2.4. Sequential Chemical Extraction Procedures

**Figure 1:**
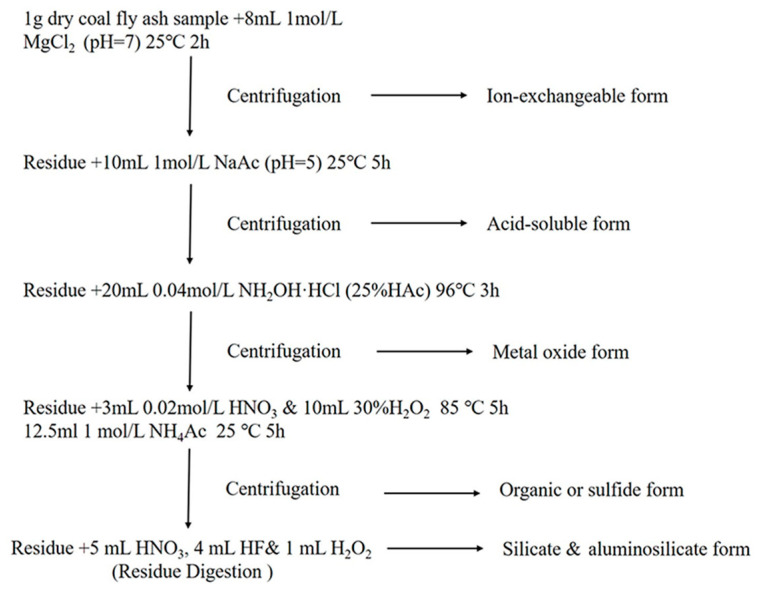
Flow chart of sequential chemical extraction procedure.

The sequential chemical extraction procedure (SCEP) is a key method for trace element speciation analysis. Based on the reported protocols [23], the REE occurrence modes are divided into five categories: ion-exchangeable, acid-soluble, metal oxides, organic or sulfide, and aluminosilicate (Figure 1).

### 2.5. Analysis Method

X-ray diffraction analysis (XRD, D8 ADVANCE, Bruker, Karlsruhe, Germany) was used to analyze the mineralogical composition of coal fly ash, with a step size of 0.01945° and a scan rate of 0.2 s/step. The XRD data were analyzed with MDI Jade 6.5. The scanning electron microscope (SEM, VEGA Compact, Tescan, Brno, Czech) was used for the morphological characterization of coal fly ash, and the metal concentrations in leachate were measured using inductively coupled plasma mass spectrometry (Agilent 7900 ICP-MS, Santa Clara, CA, USA) with an RSD of 2%. The leaching efficiency of REEs was calculated by dividing the mass of REEs in leachate by the mass of REEs in the coal fly ash. A Fourier transform infrared (FTIR) spectrometer (VERTEX 80V Bruker, Karlsruhe, Germany), with a scanning range of 4000–400 cm^−1^, was used to analyze functional groups on the surface of CFA. The cell concentration was measured by direct counting with a hemacytometer (biochrom, Ultrospec 10, Cambridge, UK). The leachate was subjected to ultrasonic treatment for 10 min before measuring cell concentration. Differences in REE bioleaching efficiency were statistically evaluated using one-way analysis of variance (ANOVA), and Tukey’s honestly significant difference (HSD) test was conducted for multiple comparisons. REE leaching efficiencies of groups with different bacterial inoculum size, pulp density and energy source were compared separately. A *p*-value < 0.05 was considered statistically significant. All data were analyzed and visualized using Excel 2021, IBM SPSS Statistics v32 and Origin 9.0.

## 3. Results and Discussion

### 3.1. Bioleaching of Zhunneng Coal Fly Ash

#### 3.1.1. Effect of Bacterial Inoculum on REEs Leaching

**Figure 2:**
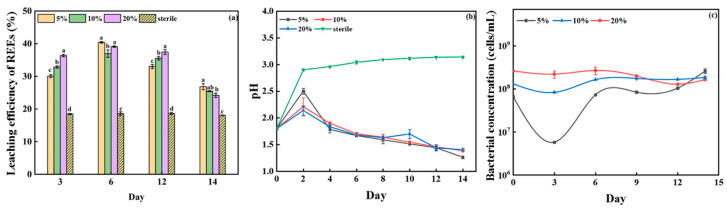
Variation in rare earth element (REE) leaching efficiency (**a**), pH value (**b**), and bacterial concentration (**c**) in bioleaching systems with different bacterial inoculum. Significant differences (*p* < 0.05) are indicated by letters.

To improve REE bioleaching efficiency, key parameters including bacterial inoculum size, pulp density and energy source were optimized. Figure 2a shows that the maximum REE leaching efficiencies occurred on day 6, which were 40.41%, 36.96%, and 39.10%, in the 5%, 10% and 20% inoculum groups, respectively. Then the REE leaching efficiency declined slightly, and reached 26.83%, 25.45%, and 24.17% on day 14, but they were still significantly (*p* < 0.05) higher than those of the control group. The decline of leaching efficiency was also observed in other studies [3,8]. It is likely due to the adsorption of REEs on *A. ferrooxidans* cells or the precipitation of REEs with silicic acid. A similar REE leaching efficiency was achieved with the 5% and the 20% inoculum size, while the group with 10% inoculum size yielded the lowest leaching efficiency.

The initial pH of the leaching systems was 1.8, which increased during the first two days and then declined until the 14th day (Figure 2b). The pH of the 5% inoculum group decreased most rapidly, reaching a final pH of 1.28, followed by the 20% inoculum group (pH 1.38). The final pH of the leaching system with a 10% inoculum size decreased to 1.40. In the control group without bacteria, pH rose rapidly to 3.0 and then increased slowly. The group with low pH showed high REE leaching efficiency. As shown in Figure 2c, the initial bacterial concentrations of the leaching system were 6.48 × 10^7^, 1.296 × 10^8^, and 2.59 × 10^8^ cells/mL in the 5%, 10%, and 20% inoculum groups, respectively. During the first three days of leaching, the bacterial population declined due to the toxic effects of the fly ash, especially in the 5% inoculation group. Subsequently, adapted bacteria began to grow and multiply, leading to an overall increase in the bacterial concentration. The most significant bacterial proliferation was observed in the 5% inoculum leaching system, and its final bacterial concentration was 2.8 × 10^8^ cells/mL.

In the early stage of bioleaching, the pH of the system increased and the bacterial concentration decreased due to the addition of coal fly ash. Coal fly ash reacted with sulfuric acid, which resulted in the pH increase. The increased pH posed challenges to the survival of bacteria. Also, the adsorption of bacteria on coal fly ash is a factor contributing to the decrease in cell concentration [8]. Due to high pH, initially the REE leaching efficiency of the 5% inoculum group was the lowest among the three experimental groups. With the progress of leaching, the cells that had developed tolerance began proliferating and metabolizing sulfur to generate sulfuric acid. The decrease in pH resulted in an increase in REE leaching efficiency. Inoculum size significantly influenced the microbial environment. High inoculum sizes introduced more cells to decompose minerals but also intensified competition among cells for essential nutrients and dissolved oxygen within the confined leaching environment [26,27]. This competition can limit the growth and metabolic activity of cells, potentially explaining the sub-optimal performance of the 20% inoculum compared with that of the 5% inoculum on day 14. An excessively low inoculum size may result in a failure of bacteria to adapt to the leaching environment. Fan & Lv [3] proposed that the optimal inoculum concentration was 3 × 10^8^ cells/mL, equivalent to the 20% inoculum in this study. We found that although the REE leaching efficiency was high in both the 5% and 20% inoculum groups, the 5% is thought to be the favorable bacterial inoculum size, considering that high inoculum concentration increases cost. However, the differences among groups may also result from operation error and measurement error of ICP-MS. A wider range of inoculum sizes deserves to be investigated in the future.

#### 3.1.2. Effect of Pulp Density on REE Leaching

**Figure 3:**
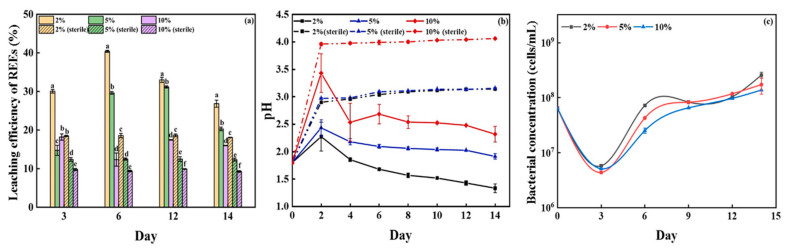
Variation in REE leaching efficiency (**a**), pH value (**b**), and bacterial concentration (**c**) in bioleaching systems with different pulp density. Significant differences (*p* < 0.05) are indicated by letters.

Pulp density affects bioleaching significantly. As shown in Figure 3a, the leaching system with 2% pulp density had the maximum REE leaching efficiency of 40.41% on day 6, significantly (*p* < 0.05) higher than the leaching systems with 5% and 10% pulp density, which achieved the maximum REE leaching efficiency of 31.17% and 17.46% on day 12. In the blank control groups, REE leaching efficiencies were 18.08%, 12.35%, and 9.27% for the 2%, 5%, and 10% pulp density groups respectively, and remained constant from day 3 to day 14.

As for pH value, the 2% pulp density group experienced the fastest decline, reaching a final pH of 1.28, followed by the 5% pulp density group, which had a minimum pH of 1.95. The 10% pulp density group showed the slowest acidification, with a minimum pH of 2.42 (Figure 3b). In the control groups, the pH value increased in the first two days and remained unchanged at ~3.0 in the 2% and 5% pulp density groups, and at 4.0 in the 10% pulp density group. Figure 3c showed that bacterial concentration decreased first and then increased. Generally, bacterial concentration was highest in the 2% pulp density group, followed by the 5% pulp density group. In the 10% pulp density group, cell concentration was the lowest. The results suggest that higher pulp density is more toxic to bacteria.

The REE leaching efficiency decreased with the increase in pulp density, consistent with previous studies [3]. The amount of coal fly ash affected the pH of the leaching system. Coal fly ash reacts with H^+^, resulting in an increase in pH [17]. The more coal fly ash is added, the higher the pH becomes. Since *A. ferrooxidans* thrives in acidic environments (pH 1.8–2.3), the high pH impaired its growth and metabolism, which directly reduced the leaching efficiency of REEs [28]. Higher pulp densities increased the viscosity of the slurry, significantly impeding oxygen mass transfer from the gas phase to the bacteria [29]. This oxygen limitation likely constrained the rate of sulfur oxidation and acid generation, further contributing to the reduced bioleaching efficiency observed at higher pulp densities. In addition, when the pulp density is high, it becomes more difficult for bacteria to access the sulfur. It is another factor that adversely affects REE bioleaching. Therefore, the REE leaching efficiency of the 2% pulp density group was the highest. However, low pulp density is unfavorable for the scale-up of the bioleaching system, as more culture medium and bacterial inoculation are needed to process a certain amount of coal fly ash. And low pulp density usually leads to low REE concentration in leachate, which increases the cost of downstream REE recovery from leachate. In the future, we will improve bacterial tolerance to high pulp density through domestication or explore column leaching and heap leaching.

#### 3.1.3. Effect of Energy Substances on REE Leaching

**Figure 4:**
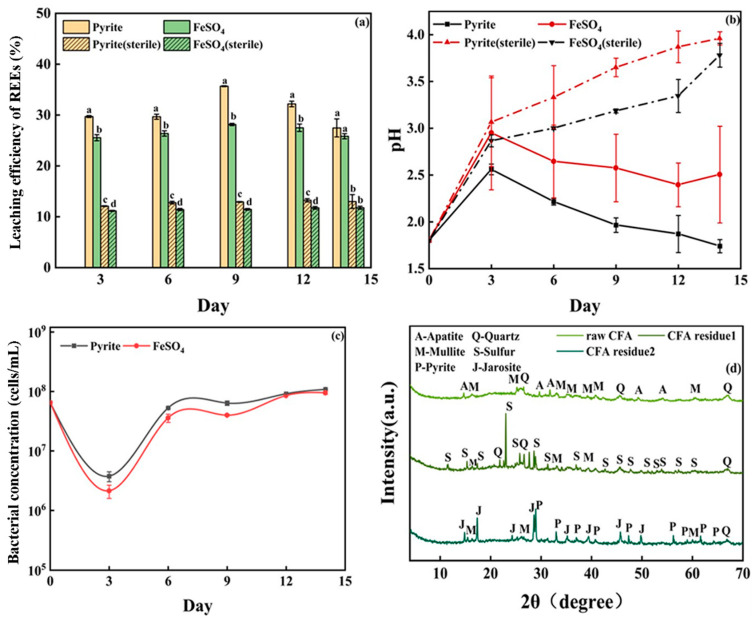
Changes in REE leaching efficiency (**a**), pH value (**b**), bacterial concentration (**c**) and composition of mineral phases (**d**) in bioleaching systems with different energy substance. CFA (coal fly ash) residue 1: bioleaching residues with elemental sulfur as energy source; CFA residue 2: bioleaching residues with pyrite as energy source. Significant differences (*p* < 0.05) are indicated by letters.

Many kinds of reduced iron or sulfur can be utilized by *A. ferrooxidans* as energy sources [19]. Energy substances significantly affect the growth and metabolism of *A. ferrooxidans* [30], but their effects on REE bioleaching remain unknown. In this study, three different energy substances were evaluated using Zhunneng coal fly ash. The results demonstrated that both pyrite and FeSO_4_ groups had a significantly (*p* < 0.05) lower REE leaching efficiency than the group with elemental sulfur (S^0^) as an energy source. Both the pyrite and FeSO_4_ groups achieved the highest REE leaching efficiency on day 9, which were 35.7% and 28.31%, respectively (Figure 4a). Also, the pyrite group exhibited a lower pH value (Figure 4b) and a slightly higher cell concentration (Figure 4c) than the FeSO_4_ group. These results were consistent with those observed with Faer coal fly ash (Figures S1–S3).

The sulfur oxidation by *A. ferrooxidans* leads to the continuous production of sulfuric acid, which maintains an erosive leaching environment and releases REEs from coal fly ash. The leaching system with pyrite as the energy material produced jarosite (Figure 4d), which is due to the oxidation of pyrite by *A. ferrooxidans* to ferrous sulfate (FeSO_4_) and sulfuric acid (H_2_SO_4_), followed by further oxidation to ferric sulfate (Fe_2_(SO_4_)_3_). In an acidic environment, the potassium ions (K^+^) in the leaching system react with ferric sulfate (Fe_2_(SO_4_)_3_) to form jarosite (KFe(SO_4_)_2_⋅12H_2_O) (Fan et al., 2019 [3]). As the amount of jarosite in the solution increased, it crystallized into a water-insoluble solid, hindering further dissolution of coal fly ash. Jarosite was also observed in the FeSO_4_ group (Figure S5). However, no jarosite was observed in the group with S^0^ as the energy source, explaining its REE leaching efficiency being the highest among the three groups. In addition, with pyrite or FeSO_4_ as energy source, additional Fe^3+^ is introduced into the leachate as an impurity, which would cause difficulties in subsequent REE separation and purification.

The pyrite system exhibited a higher REE leaching efficiency than the FeSO_4_ system. Pyrite was oxidized into H_2_SO_4_ and Fe^3+^ by *A. ferrooxidans*. It created more favorable conditions, such as pH and redox potential, for microbial growth and coal fly ash decomposition, enhancing the REE bioleaching [3]. In contrast, although the FeSO_4_ system experienced a slight decrease in pH due to Fe^2+^ oxidation to Fe^3+^ and subsequent hydrolysis, the pH was still relatively high, resulting in a lower leaching efficiency of REEs. With FeSO_4_ as the energy source, REE leaching efficiency was higher in the experimental group than in the abiotic control group, but the bioleaching mechanism remains unclear; extracellular polymeric substances probably play a role in the process [30].

In summary, the favorable bacterial inoculum and pulp density were 5% and 2%, respectively. Among the bioleaching systems using elemental sulfur, pyrite, and FeSO_4_ as energy sources, the system with sulfur achieved the highest leaching efficiency of REEs, followed by the system using pyrite. In this study, the REE bioleaching efficiency reached 40.41%, which is comparable to the REE extraction efficiency in other studies. The REE chemical leaching efficiency of raw coal fly ash was around 30–60% in most studies [23]. As for bioleaching, 30.91% of REEs were leached by *A. niger*, and *Acidithiobacillus thiooxidans* extracted 38.3–87.1% of the REEs from unpretreated coal fly ash (Table S1).

### 3.2. Selectivity

**Figure 5:**
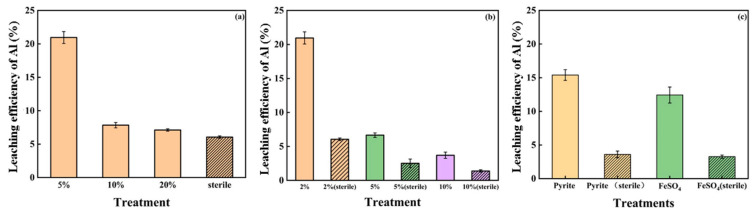
The impact of bacterial inoculum size (**a**), pulp density (**b**) and energy substances (**c**) on aluminum (Al) bioleaching.

During bioleaching of coal fly ash, aluminum (Al) was also dissolved, which is affected by bacterial inoculum, pulp density and energy source. Figure 5a showed that the system with 5% bacterial inoculum had the highest Al leaching efficiency (20.32%), followed by the groups with 20% inoculum (7.55%) and 10% inoculum (7.24%). The leaching efficiencies of Al exhibited an inverse relationship with pulp density (Figure 5b). Al bioleaching efficiency in the group with pyrite as the energy source was lower than that in the elemental sulfur group, but higher than that in the FeSO_4_ group (Figure 5c and Figure S6). Leaching efficiencies in all experimental groups were higher than those in the blank control groups. These results are similar to those observed in REE bioleaching.

Selectivity means that specific elements are more easily solubilized and extracted during the leaching process, while reducing unnecessary by-products. In this study, REEs exhibited a leaching efficiency of 40.41%, which is 1.99 times that of Al, demonstrating that *A. ferrooxidans* has selectivity for REEs. This also holds true for the Faer coal fly ash (Figure S6). Furthermore, the selectivity is mainly for LREE, as LREE had higher leaching efficiencies than HREE (Figure S7). The selectivity for REEs is better than that reported in a previous study [3], in which 91.2% Al and 63.4% Ce were leached by *A. ferrooxidans* from coal fly ash pretreated via alkaline roasting. The alkaline roasting pretreatment transformed refractory aluminosilicate (mullite and sillimanite) into nepheline (NaAlSiO_4_), which exhibits significantly higher reactivity towards acids including biogenic sulfuric acid. After alkaline roasting, the acid leaching efficiency of Ce increased slightly (by ~6%) but the acid leaching efficiency of Al increased significantly (by ~60%), thereby reducing the selectivity for REEs. In addition, the surface energy of nepheline is higher than that of mullite, and thus more bacterial cells are likely to adsorb onto the surface of nepheline [21]. This increased adsorption enhances the bioleaching of Al.

In bioleaching, the adsorption of microorganisms onto mineral surfaces is essential for their oxidizing action. Research has shown that microorganisms preferentially attach to regions rich in specific metal elements (such as REEs and heavy metals), as these metal elements can serve as electron acceptors for bacterial metabolism [19]. The extracellular polymeric substances (EPSs) secreted by bacteria are enriched with anionic functional groups, such as sulfate and carboxyl groups, which exhibit higher binding affinity for REE^3+^ ions compared to Al^3+^ [31,32].

Moreover, the high selectivity of bioleaching for REEs is also related to pH and temperature [33]. Under elevated H^+^ concentrations, the activation energy barrier governing aluminosilicate dissolution is forcibly bypassed through proton saturation effects, which promotes the dissolution of Al [34]. In other words, excess input of H^+^ leads to total disintegration of the mineral structure and non-selective release of all metals. In this study, the pH is significantly higher than that in chemical leaching [35], which is a factor contributing to selectivity for REEs. Bioleaching temperature is much lower than that of chemical leaching, which is another reason for high selectivity. In summary, direct bioleaching of coal fly ash is highly selective for REEs, which will simplify subsequent separation and purification processes and lower the cost.

### 3.3. Faer Coal Fly Ash Bioleaching

**Figure 6:**
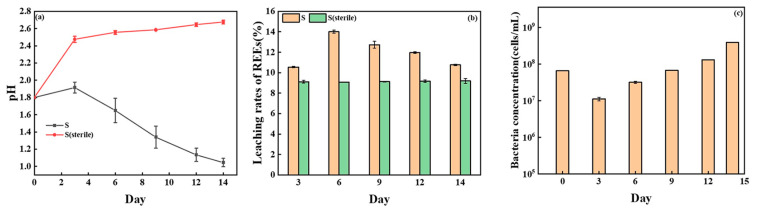
Changes in pH value (**a**), leaching efficiency of REEs (**b**) and bacterial concentration (**c**) of Faer coal fly ash bioleaching systems.

The composition and structure of coal fly ash are key factors affecting REE bioleaching. Here, two kinds of coal fly ash with different origins and combustion methods were investigated. In the Faer coal fly ash bioleaching system, pH decreased from 1.80 to 1.01, and REE leaching efficiency reached 12.93% on day 6 (Figure 6). In Faer CFA, the leaching efficiencies of individual REEs are similar, except for Lu. By contrast, the leaching efficiencies vary a lot among individual REEs in Zhunneng CFA (Figure S7). The REE leaching efficiency in experimental groups was significantly higher than that of the abiotic control group (pH 1.80) and the control group with 0.31 mol/L sulfuric acid (Figure S8), demonstrating that bioleaching is superior to sulfuric acid leaching under specific conditions. Due to the toxicity of coal fly ash, bacteria exhibited an initial 3-day adaptation phase followed by proliferation. Under the same leaching conditions, the REE leaching efficiency of Zhunneng coal fly ash was about 27% higher than that of Faer coal fly ash, consistent with that of hydrochloric acid leaching [23]. It holds true for all the individual REEs (Figure S7). Faer coal fly ash originates from a pulverized coal furnace (PC), while Zhunneng coal fly ash is produced by a circulating fluidized bed (CFB). The two kinds of coal fly ash are different in morphology, chemical composition, and mineralogical composition [36]. These differences affect REE recovery from coal fly ash.

### 3.4. The Influence Mechanisms of Coal Fly Ash Type on REE Bioleaching

#### 3.4.1. Morphological Analysis

**Figure 7:**
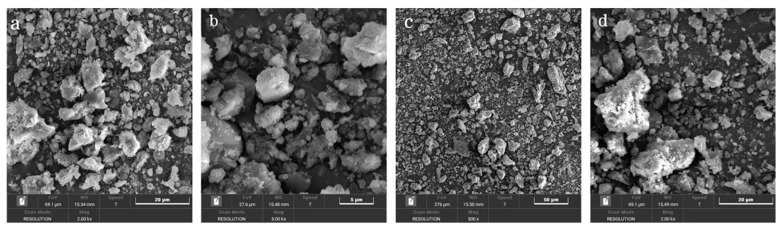
Scanning electron microscopy of raw Zhunneng coal fly ash samples (**a**,**b**) and residues after bioleaching (**c**,**d**). (ref. [38]).

**Figure 8:**
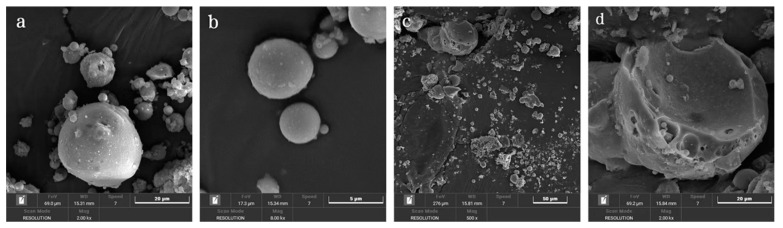
Scanning electron microscopy of raw Faer coal fly ash samples (**a**,**b**) and residues after bioleaching (**c**,**d**).

To reveal the bioleaching mechanism of two kinds of coal fly ash, morphological characteristics, mineral phases, and REE occurrence modes were analyzed. SEM analysis of the raw coal fly ash samples and their bioleaching residues revealed that the surface morphologies of the two coal fly ash samples are significantly different (Figure 7 and Figure 8). The raw Zhunneng CFA from circulating fluidized bed exhibited characteristics of irregular particles, fragmentation, and agglomeration into larger particles (Figure 7a,b), which is conducive to bacterial adsorption. After bioleaching, the coal fly ash surface became more fragmented, with visible fine particles and etching pits that exposed internal spaces (Figure 7c,d). In contrast, there were mainly spherical glass beads in raw Faer CFA (Figure 8a,b), which is the typical characteristic of coal fly ash generated from a pulverized coal furnace [23]. The temperature in a pulverized coal furnace is around 1400–1500 °C, higher than that in a circulating fluidized bed. Under a high temperature, minerals in coal melt and form spherical glass microbeads because of surface tension. The spherical shape indicates Faer CFA has a small specific surface area, which limits its contact with bacteria and results in a low REE leaching efficiency. Similar textural characteristics were also observed in other studies. For example, Zhang et al., [37] observed plate-like particles with needled materials in one CFA sample and spherical shaped particles and irregular debris in another CFA sample.

The bioleaching process caused varying degrees of damage to the spherical structure of the Faer coal fly ash, resulting in the formation of holes and cracks on the surfaces (Figure 8c,d). The disruption of the fly ash structure exposed the encapsulated rare earth metals. Sulfuric acid produced by *A. ferrooxidans* can dissolve certain minerals in the coal fly ash and create cavities. Additionally, other microbial metabolites, such as organic acids and extracellular polymeric substances, can form complexes with metal elements in the coal fly ash, enhancing metal leaching [18,31]. Together, these processes created voids and cracks on CFA particle surfaces.

#### 3.4.2. Mineralogical Composition

**Figure 9:**
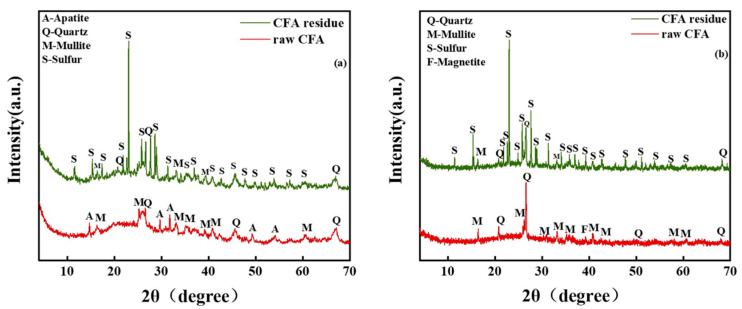
Changes in the composition of mineral phases before and after bioleaching of Zhunneng (**a**) and Faer (**b**) coal fly ash. Raw CFA: original coal fly ash without any treatment; CFA residue: bioleaching residues with sulfur as energy source.

The mineralogical composition of the two coal fly ash samples is different. As shown in Figure 9, the main minerals in the raw Zhunneng coal fly ash are quartz, mullite, and apatite. The main components of Faer coal fly ash are quartz, magnetite, and mullite. The diffraction intensity of quartz and mullite in Faer is significantly higher than that in Zhunneng coal fly ash. The hump at the 2θ of 20–30° indicates the presence of an amorphous phase, which appeared in both coal fly ash samples. In the bioleaching system with sulfur as the energy source, the main components in both coal fly ash residues are unused sulfur, mullite, and quartz.

The acidic environment created by the metabolism of *A. ferrooxidans* is the main factor driving the mineral phase changes during bioleaching. In Zhunneng CFA, apatite reacts with hydrogen ions (H^+^), resulting in the breaking of calcium-oxygen bonds (Ca-O) and formation of PO_4_^3−^. Phosphate (PO_4_^3−^) also reacts with hydrogen ions (H^+^) to form hydrogen phosphate (HPO_4_^2−^) or undergoes further protonation to form dihydrogen phosphate (H_2_PO_4_^−^). These protonated phosphate species subsequently formed complexes with metal ions in the solution [39]. These reactions lead to the disruption of the apatite structure. The dissolution of magnetite also depends on this acidic environment. For mullite and quartz, H^+^ preferentially attacks the oxygen atoms on the mineral surface, initiating the dissolution process via cleavage of the Si-O and Al-O bonds [40]. This is supported by FTIR analyses, which showed a decrease in the intensity of the characteristic peak at 1095 cm^−1^ (Si-O stretching vibration) in the leaching residue of Zhunneng coal fly ash (Figure S9). However, mullite and quartz have strong chemical bonds, and are more stable than other minerals in coal fly ash and thus remain in the bioleaching residue.

The stability of minerals directly influences the extraction of REEs. Mineral phases such as mullite and quartz are chemically stable, and the REEs within them are difficult to leach [41]. There were a lot of mullite and quartz in Faer coal fly ash, resulting in its poor REE extraction. The high REE leaching efficiency of Zhunneng coal fly ash is attributed to the presence of apatite, which undergoes rapid dissolution under acidic conditions, directly releasing substantial amounts of REEs. Moreover, dissolved REEs may be derived from amorphous silicate, which is less stable than crystalline silicate and more easily decomposed by microorganisms [42]. The mineralogical composition of coal fly ash significantly affects the leaching efficiency of REEs, providing a key theoretical basis for developing extraction strategies based on mineralogical characteristics.

#### 3.4.3. Occurrence Modes of REEs

**Figure 10:**
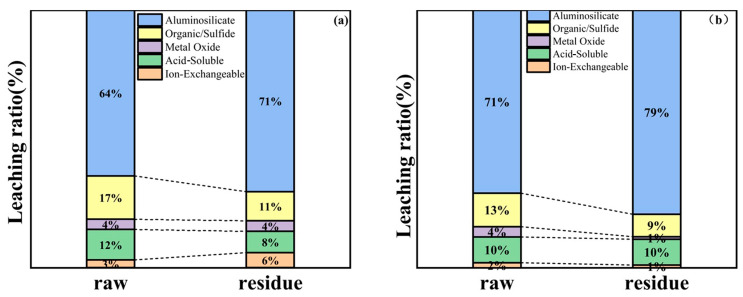
Results of the sequential chemical extraction procedure of Zhunneng (**a**) and Faer (**b**) coal fly ash raw samples and bioleaching residues.

The sequential chemical extraction procedure is widely used to understand the occurrence modes of trace elements in minerals and their geochemical properties. The sequential chemical extraction analyses of Zhunneng and Faer coal fly ash revealed that the silicate or aluminosilicate form predominated, accounting for 64% and 71% of the total REEs, respectively (Figure 10). Notably, more REEs were associated with aluminosilicate in Faer coal fly ash than in the Zhunneng sample. The organic/sulfide form constituted 17% and 13% of the total REEs for the Zhunneng and Faer samples, respectively, followed by the acid-soluble fraction (~10%). The acid-soluble REEs in coal fly ash are probably REE oxides and carbonates (Liu et al., 2019 [41]). A small portion of REEs occurred in ion-exchangeable or metal oxide fractions (<5%) in both samples. Zhunneng coal fly ash contained more organic/sulfide and acid-soluble REEs than the Faer samples, suggesting that its REEs are more easily extracted.

After bioleaching, the sequential chemical extraction analyses of the residues indicated that the aluminosilicate fraction increased to 71% and 79% for Zhunneng and Faer coal fly ashes, respectively. Conversely, the organic/sulfide form decreased from 17% to 11% in the Zhunneng coal fly ash and from 13% to 9% in the Faer sample. The acid-soluble fraction decreased by 4% in the Zhunneng sample, but remained unchanged in Faer coal fly ash after bioleaching. The metal oxide form in Faer coal fly ash dropped from 4% to 1%. No significant change was observed in other fractions. The results are consistent with previous studies. Liu and Huang [41] demonstrated that during acid leaching, rare earth oxides and carbonates dissolve at pH 5.5–4.0, apatite at pH 3.5–2.0, and REEs bearing phosphate and hematite dissolve at pH < 1.5, while aluminosilicates remain intact. In this study, *A. ferrooxidans* generated sulfuric acid, achieving a final pH < 1.5, which led to the dissolution of metal oxides. Additionally, SO_4_^2−^ may precipitate with Ca^2+^, covering coal fly ash surfaces and limiting sulfuric acid contact, potentially increasing the relative proportion of ion-exchangeable REEs [39].

The organic/sulfide phase likely refers to REEs enriched in unburnt carbon or sulfide. The metabolism of *A. ferrooxidans* could increase redox potential, which may facilitate the leaching of REEs associated with the organic/sulfide phase. During bioleaching, REEs encapsulated in aluminosilicates are barely released, while other forms of REEs are leached out, resulting in a higher relative proportion of the aluminosilicate fraction in the residue. Particularly, more than 60% of the REEs could be bioleached from Zhunneng coal fly ash, but the aluminosilicate fraction accounted for 64% of total REEs and other fractions accounted for only 36%, suggesting that part of the aluminosilicate-associated REEs was dissolved, especially the amorphous phase. The occurrence modes of REEs in the two types of coal fly ash are different, which ultimately results in differences in leaching efficiency.

Overall, different combustion methods lead to the differences between the two coal fly ashes in morphology, mineral phase, and REE occurrence modes, thus affecting bioleaching of REEs. Faer coal fly ash had a spherical shape and a smooth surface, contained more quartz and mullite, and had more REEs associated with aluminosilicate than the Zhunneng sample. These characteristics result in a poor REE extraction efficiency. Zhunneng coal fly ash displayed an irregular fragmented shape, which is conducive to the adsorption of bacteria. Furthermore, a greater proportion of REEs occurred as organic/sulfide and acid-soluble forms in Zhunneng coal fly ash, which are easily dissolved by *A. ferrooxidans*. The in-depth understanding of the mechanisms underlying bioleaching provides a theoretical foundation for its industrial application in REE recovery from coal fly ash.

## 4. Conclusions

In this study, the recovery of REEs from coal fly ash was achieved via bioleaching. The favorable bioleaching conditions were obtained as follows: 2% pulp density, 5% bacterial inoculum size, and elemental sulfur as the energy source. Compared with the Faer coal fly ash, the rare earth elements (REEs) in the Zhunneng coal fly ash exhibited a higher leaching efficiency. Further investigation revealed that Faer coal fly ash contains more quartz and mullite, which are more difficult for bacteria to solubilize than amorphous silicate. Also, Faer coal fly ash contains more REEs associated with aluminosilicate, while Zhunneng coal fly ash contains more organic/sulfide-bound and acid-soluble REEs, which are easily bioleached. It is pointed out for the first time that the direct bioleaching method has high selectivity for REEs, as REE leaching efficiency was significantly higher than that of aluminum. This study provides new insights into the bioleaching mechanisms of two typical types of coal fly ash, which lay the foundation for low-cost, green, and classified recycling of REEs from coal fly ash.

## Data Availability

The original contributions presented in this study are included in the article/Supplementary Material. Further inquiries can be directed to the corresponding author.

## Supplementary Materials

The following supporting information can be downloaded at: https://www.mdpi.com/article/10.3390/ma19132861/s1, Figure S1. Variation of pH in Faer coal fly ash bioleaching system with three different energy substances. Figure S2. The leaching efficiency of REEs in Faer coal fly ash bioleaching system with three different energy substances. Figure S3. Changes in bacterial concentration in Faer coal fly ash bioleaching system with three different energy substances. Figure S4. Changes in the composition of mineral phases before and after bioleaching of Faer coal fly ash. Raw CFA: original coal fly ash without any treatment; CFA residue 1: bioleaching residues with sulfur as energy source; CFA residue 2: bioleaching residues with pyrite as energy source. Figure S5. Changes in the composition of mineral phases before and after bioleaching of Zhunneng (a) and Faer (b) coal fly ash. Raw CFA: original coal fly ash without any treatment. CFA residue: bioleaching residues with FeSO_4_ as energy source. Figure S6. The leaching efficiency of Al in Faer coal fly ash bioleaching system with three different energy substances. Figure S7. Leaching efficiency of individual rare earth elements, light rare earth elements (LREE) and heavy rare earth elements (HREE) in Zhunneng (a) and Faer (b) coal fly ash. Figure S8. The REEs leaching efficiency of Zhunneng and Faer CFA under sulfuric acid leaching and bioleaching. Figure S9. FTIR spectra results of Zhunneng (a) and Faer (b) coal fly ash raw samples and bioleaching residues. Table S1. A summary of bioleaching efficiency of REEs in publications. References [43,44] are cited in the Supplementary Materials.
