# Supplementary material for: Recovery of Rare Earth Elements from Different Types of Coal Fly Ash by Direct Bioleaching
Source: Materials (Basel). 2026 Jul 4;19(13):2861. doi: 10.3390/ma19132861 (PMC13362778; doi:10.3390/ma19132861)
Supplement: Supplementary file 1 [file materials-19-02861-s001.zip › materials-4354310-supplementary.pdf]

Recovery of rare earth elements from different types of coal fly ash by direct bioleaching – Supplementary materials

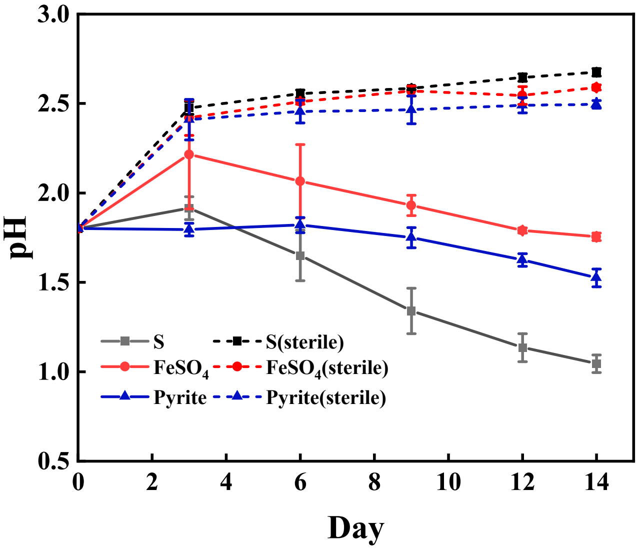

Figure S1. Variation of pH in Faer coal fly ash bioleaching system with three different energy substances.

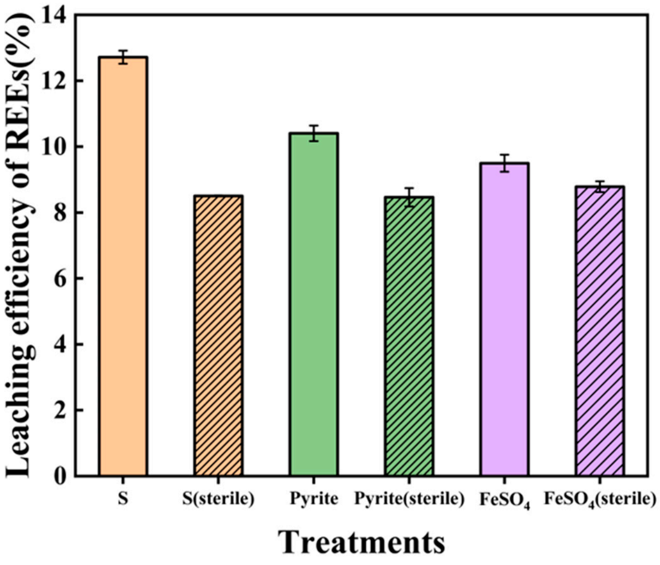

Figure S2. The leaching efficiency of REEs in Faer coal fly ash bioleaching system with three different energy substances.

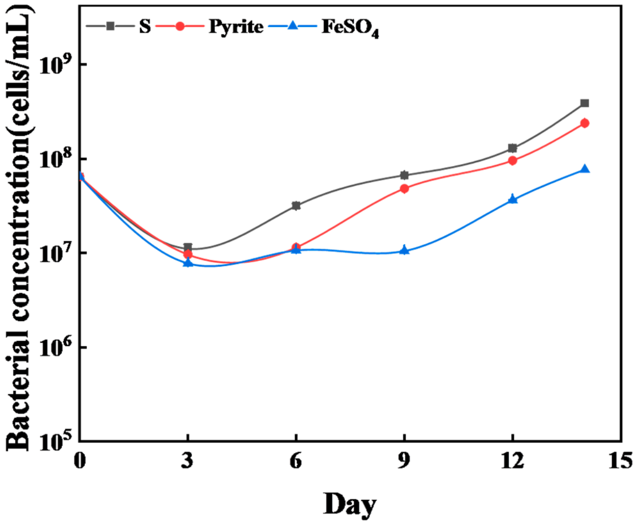

Figure S3. Changes in bacterial concentration in Faer coal fly ash bioleaching system with three different energy substances.

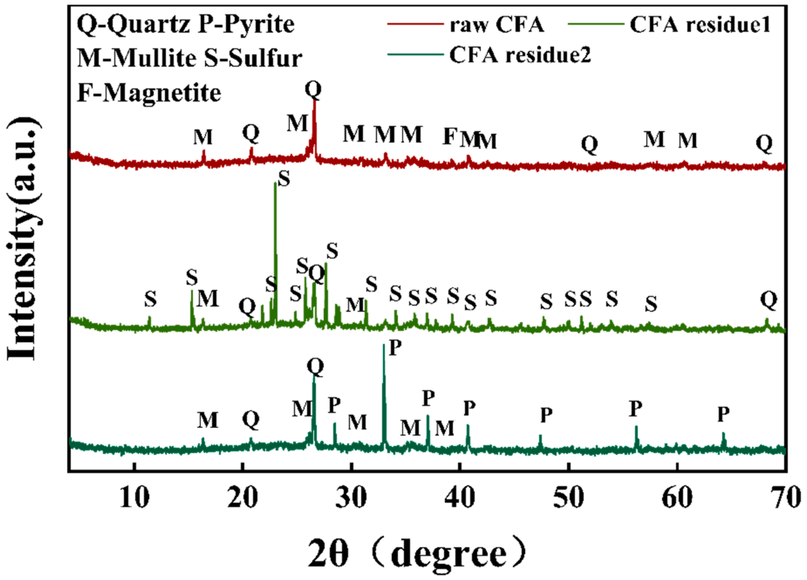

Figure S4. Changes in the composition of mineral phases before and after bioleaching of Faer coal fly ash. Raw CFA: original coal fly ash without any treatment; CFA residue 1: bioleaching residues with sulfur as energy source; CFA residue 2: bioleaching residues with pyrite as energy source.

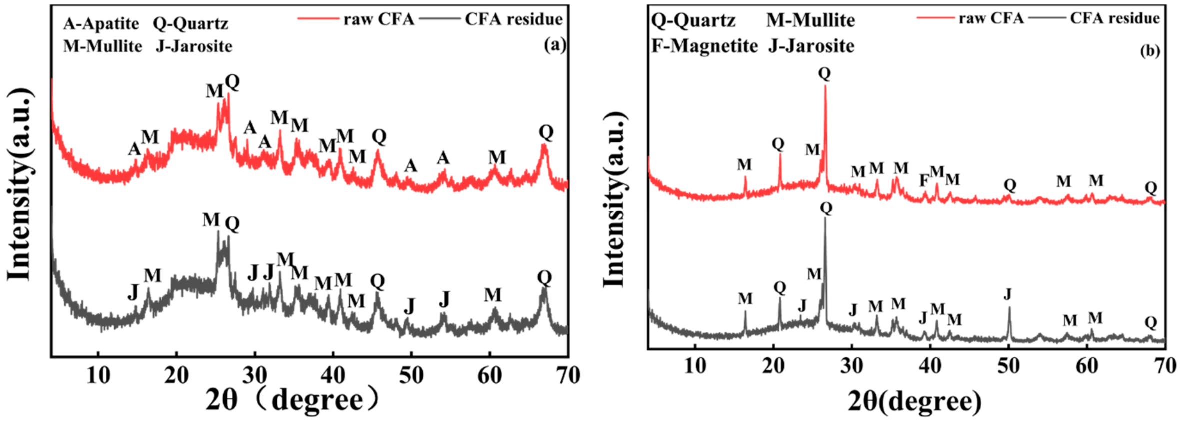

Figure S5. Changes in the composition of mineral phases before and after bioleaching of Zhunneng (a) and Faer (b) coal fly ash. Raw CFA: original coal fly ash without any treatment. CFA residue: bioleaching residues with $FeSO_4$ as energy source.

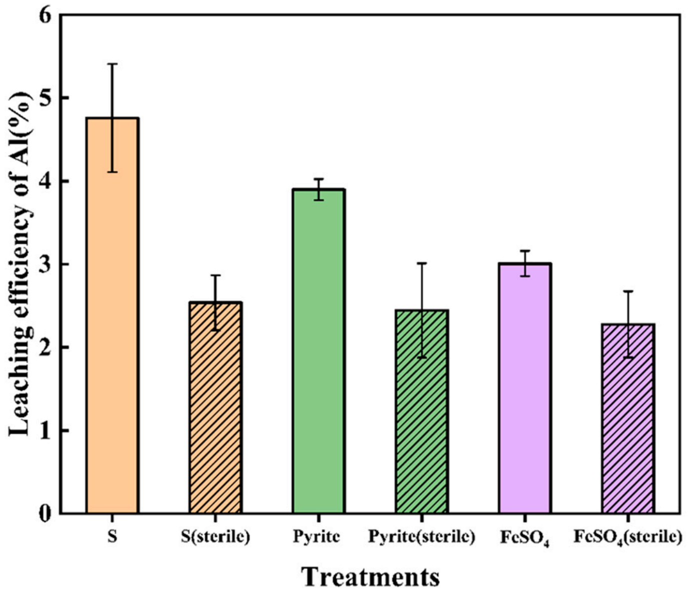

Figure S6. The leaching efficiency of Al in Faer coal fly ash bioleaching system with three different energy substances.

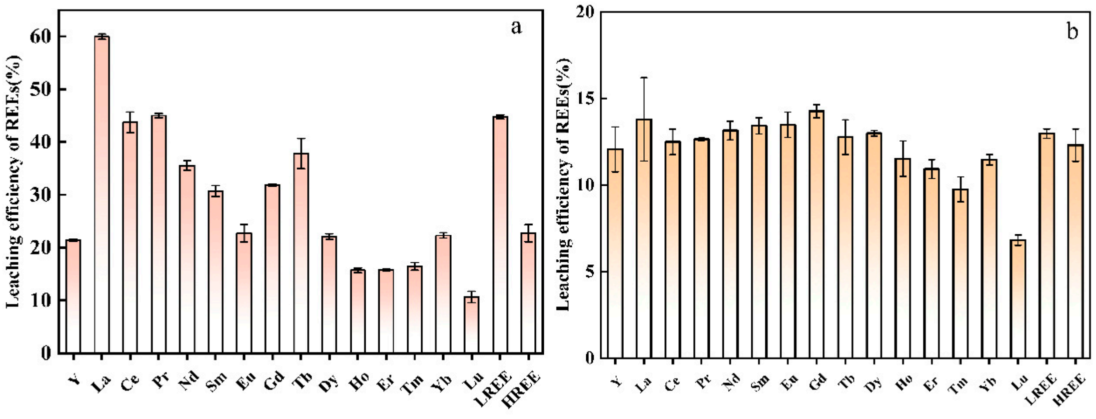

Figure S7. Leaching efficiency of individual rare earth elements, light rare earth elements (LREE) and heavy rare earth elements (HREE) in Zhunneng (a) and Faer (b) coal fly ash.

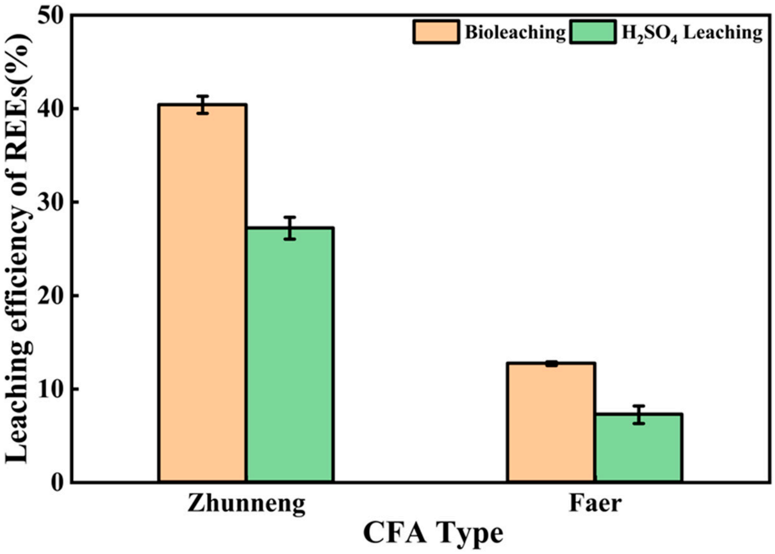

Figure S8. The REEs leaching efficiency of Zhunneng and Faer CFA under sulfuric acid leaching and bioleaching.

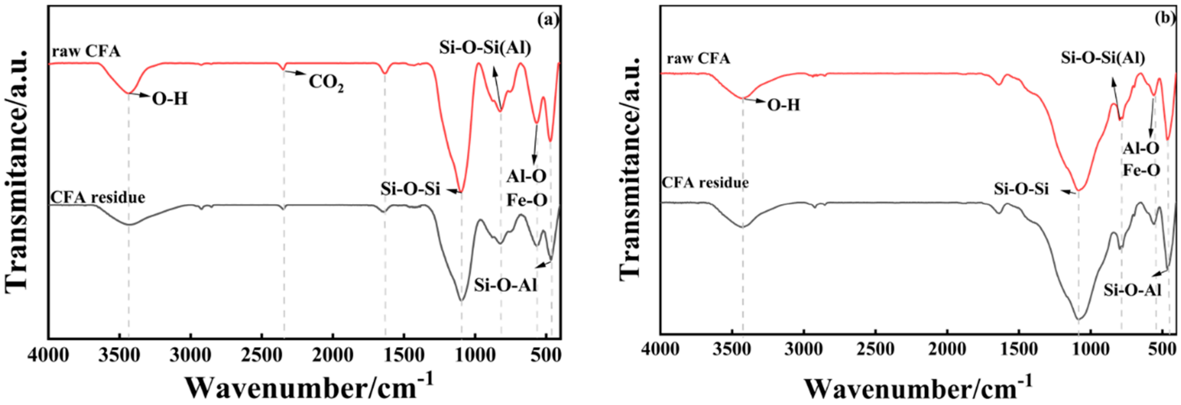

Figure S9. FTIR spectra results of Zhunneng (a) and Faer (b) coal fly ash raw samples and bioleaching residues.

Table S1. A summary of bioleaching efficiency of REEs in publications.

Microbial species	Pulp density	Pretreatment	Leaching time	Leaching efficiency	References
Acidithiobacillus ferrooxidans	30%	No	3 d	13-14%	Zhang et al., 2021
Aspergillus niger	5%	No	3 d	40-45%	Khan et al., 2026
Aspergillus niger	5%	No	3 d	30.91%	Ma et al., 2023
A. ferrooxidans	2%	Na_2CO_3 roasting	15 d	63.4% Ce	Fan et al., 2019
Acidithiobacillus thiooxidans	10%	No	21 d	38.3% – 87.1%	Su et al., 2020

		Hydrothermal alkali	21 d	70.0% – 97.6%	
Candida bombicola				27.3% – 67.7%	
Phanerochaete chrysosporium	1%	No	6 h	21.8% – 50.6%	Park et al., 2019
Cryptococcus curvatus				19.5% – 56.1%	

References

Fan, X. L., Lv, S. Q., Xia, J. L., Nie, Z. Y., Zhang, D. R., Pan, X., Zhao, Y. D. Extraction of Al and Ce from coal fly ash by biogenic Fe^{3+} and H_2SO_4. Chemical Engineering Journal 370, 1407-1424

Khan, Asghar; LIAQAT, Nabeel; YU, Xiong Bill. Biogenic pathways for rare-earth recovery from coal fly ash via fungal mineralization. Environmental Management: Engineering and Technology, 2026, 100004.

Ma, Juanjuan, et al. Bioleaching rare earth elements from coal fly ash by *Aspergillus niger*. Fuel, 2023, 354: 129387.

Park, Stephen; LIANG, Yanna. Bioleaching of trace elements and rare earth elements from coal fly ash. International Journal of Coal Science & Technology, 2019, 6.1: 74.

Su, H., Tan, F., Lin, J. An integrated approach combines hydrothermal chemical and biological treatment to enhance recycle of rare metals from coal fly ash. Chemical Engineering Journal 395, 124640

Zhang, Zongliang, et al. Recovery and enhanced upgrading of rare earth elements from coal-based resources: Bioleaching and precipitation. Minerals, 2021, 11.5: 484.
